# Maternal Characteristics Are Associated with Child Dietary Diversity Score, in Golina District, Northeast Ethiopia: A Community-Based Cross-Sectional Study

**DOI:** 10.1155/2020/6702036

**Published:** 2020-09-22

**Authors:** Getahun Fentaw Mulaw, Fentaw Wassie Feleke, Seteamlak Adane Masresha

**Affiliations:** ^1^Public Health Department, College of Health Sciences, Samara University, Samara, Afar, Ethiopia; ^2^Public Health Department, College of Health Sciences, Woldia University, Woldia, Ethiopia

## Abstract

**Background:**

Dietary diversity is part of the set of indicators developed to assess infant and young child feeding practices. In developing countries, only a quarter of children met the required minimum dietary diversity. In Ethiopia, only 14% of children aged 6–23 months met the minimum dietary diversity score, with regional variation. Therefore, this study aimed to assess dietary diversity score and associated factors among children aged 6–23 months in Golina district, Afar region, Ethiopia.

**Method:**

A community-based cross-sectional study was conducted among 345 study participants from February 15 to March 30, 2017, in Golina district, Afar, Northeast Ethiopia. The study kebeles were selected randomly and the study subjects were selected using a cluster sampling technique. The child dietary diversity score was determined by the WHO child dietary diversity score scale, using a 24-hour dietary recall method, and data were collected using an interviewer-administered questionnaire. Multivariable logistic regression was used to identify predictor variables, and the level of significance was determined at *P* value <0.05.

**Result:**

This study revealed that children who met the required minimum dietary diversity score were 35.1% (95% CI, (30%–40%)). Children whose mothers have not attended formal education were 3.042 times (AOR = 3.042 95% CI: (1.312–7.052)) less likely to meet the minimum dietary diversity score than children whose mothers have attended secondary and above. Children whose mothers had normal BMI were 51.2% (AOR = 0.488, 95% CI: (0.259–918)) and 68.1% (AOR = 0.319, 95% CI: (0.119–0.855)) more likely to meet the minimum dietary diversity score than children whose mothers' BMI was underweight and overweight, respectively.

**Conclusion:**

Maternal characteristics (educational status and nutrition status) were found to be associated with their child's dietary diversity score. This study also revealed that children who met the minimum dietary diversity score were few. Therefore, the increased emphasis on the importance of the education of girls (future mothers) and nutrition counseling for girls/women who currently have received little education on ways to improve the family and child dietary feeding practice is needed.

## 1. Introduction

Proper infant and young child feeding practice within the first two years of life is crucial for optimal child growth, development, better health, and preventing chronic degenerative disease [1]. According to the World Health Organization (WHO), introducing optimal complementary foods exactly at six months of age is vital to fulfilling children's nutritional requirements [2, 3].

Optimal complementary food is explained by fulfilling minimum dietary diversity, meal frequency, and an acceptable diet. Minimum dietary diversity refers to the proportion of children aged 6–23 months who received, on a preceding day, at least four or more varieties of foods from the seven standard food groups recommended by the WHO, without imposing a minimum intake restriction [2, 4–6].

Greater than two-thirds of malnutrition-related child deaths are associated with inadequate dietary feeding practices during the first two years of life. In developing countries, only a quarter of children met the required minimum dietary diversity [7, 8]. Inappropriate child feeding practices contribute to poor physical growth, including irreversible outcomes of stunting, poor cognitive development, increased risk of infectious diseases, and mortality [2, 9, 10].

Globally, there are efforts to improve the nutritional status of children by implementing different programs like the zero hunger draft [11], scaling up nutrition (SUN) movement [12–14], and sustainable development goals (SDGs) [15]. The World Health Assembly has a target for reducing stunted children by 40% in 2025 [9]. At least 12 of the 17 SDGs are highly related to nutrition [16, 17].

Currently, the Ethiopian government also set targets to improve the nutritional status of children through the National Nutrition Program (NNP-II) [18], Health Sector Transformation Plan (HSTP) [19], and Health Extension Program (HEP) [20]. Ethiopia also declared to end child malnutrition by 2030 [21, 22]. Despite the above efforts, currently, 38% of children are stunted, 10% are wasted, and 24% are underweight. In Ethiopia, 14%, 45%, and 7% of children aged 6–23 months have met the minimum dietary diversity, minimum meal frequency, and minimum acceptable diet, respectively [23].

There are no documented studies on the dietary diversity feeding and associated factors among children aged 6–23 months in Northeast Ethiopia. Therefore, this study aimed to assess child dietary diversity score and associated factors among children aged 6–23 months in Golina district, Northeast Ethiopia.

## 2. Methods and Materials

### 2.1. Study Design, Setting, and Participants

A community-based cross-sectional study was conducted in Golina district from February 15 to March 30, 2017. The district is found in the pastoralist region of Afar regional state, located 691 km northeast of Addis Ababa, the capital city of Ethiopia, and 220 km northwest of Samara, the capital city of the region. According to the projection of the 2007 census, the total population of the district was estimated to be 56,929; of which 5,727 were under-five children [24]. In the district, there were 1 general hospital, 2 health centers, and 10 health posts. Participants from the randomly selected kebeles were considered as the study population. Those who gave recently up-to-date information about their index children aged 6–23 months were the study unit.

### 2.2. Sample Size and Sampling Technique

The minimum sample size for each specific objective was separately calculated using a single population proportion formula (Table 1) and double population proportion formula using EPI Info 7.1 (Table 2).

Finally, the required size was taken as 347, since this was found as the largest from all calculated sample sizes (Tables 1 and 2). Four kebeles were selected randomly from a total of eight kebeles in Golina district. The sample size was allocated proportionally for each selected kebele, based on the number of children less than two years of age. Then study units were selected using a simple random sampling method.

### 2.3. Data Collection Tools and Instruments

Data were collected using a semistructured questionnaire via face-to-face interviews with mothers of children aged 6–23 months. The questionnaire was prepared in English and translated into the local language and retranslated back to English to check the consistency. The Food and Agriculture Organization (FAO) meal frequency questionnaire was used to determine the weekly food group consumption of the children [2].

Pretesting was done on 10% of the calculated sample size in nonselected kebeles, and then the necessary modification was done. Anthropometric measurements, such as the mother's height and weight, were performed following WHO standard procedures. The mother's weight was measured using UNICEF electronic weighing scales. The weighing scales were calibrated and standardized using a 5 kg standard weight, every morning before the actual measurements. The children's age was determined using written official documents like vaccination cards. For those who had no written evidence of birthdate, a preprepared local calendar was used by cross-checking with the other family members. The children's vaccination status was assessed by card, BCG scar, and mother recalled.

Four data collectors and two supervisors, who were fluent in the local language, were employed. Two days of training, on the study tool and anthropometric measurement procedures, were given for data collectors and supervisors. When households found having two or more eligible children, one of them was selected randomly via the lottery method.

### 2.4. Operational Definitions

- Minimum child dietary diversity (MCDD): when a child gets at least 4 from the 7 WHO recommended food groups, regardless of the portion size and meal frequency [2].
- Minimum meal frequency (MMF): calculated by grouping child age into two (6–8 months and 9–23 months). The acceptable MMF of children aged 6–8 months is considered as two or more times per 24 hours while for children aged 9–23 months it is three or more times [2].
- Minimum acceptable diet (MAD): the percentage of breastfed children 6–23 months of age who had at least the minimum dietary diversity and the minimum meal frequency during the previous day, and nonbreastfed children 6–23 months of age who received at least two milk feedings and had at least the minimum dietary diversity not including milk feeds and the minimum meal frequency during the previous day [2].

### 2.5. Data Processing and Analysis

Data were edited, cleaned, coded, and entered into EPI Data 4.2 and exported to SPSS version 22 for analysis. Characteristics of study participants were summarized using descriptive statistics. Multivariable logistic regression was used to identify independent predictors of childhood dietary diversity. The strength of association was measured through the odds ratio at a 95% confidence interval. Predictor variables with the *P* value <0.25 at bivariable analysis were included in the multivariable logistic regression model and analyzed using the backward logistic regression model. Statistical significance was declared at a *P* value <0.05.

Multicollinearity was checked using standard error as a cutoff of 2, but no variables were found. The percentage of the model accurately classified was 70.7%, with Hosmer and Lemeshow goodness-of-fit test *P* value = 0.857, indicating the model fits well.

## 3. Result

### 3.1. Sociodemographic Characteristics of Study Participants

A total of 345 study participants were included, with a 97% response rate. Nearly 264 (76.5%) were lived in rural areas, and 284 (82.3%) were of Afar ethnicity. Almost quarters (254 (73.6%)) of mothers had not attended formal education. More than half (180 (52.2%)) of children were female (Table 3).

### 3.2. Maternal Health and Nutritional Status Characteristics

More than half (202 (58.5%)) of mothers were not attended ANC, and 267 (76.8%) of them were given birth at home (Table 4).

### 3.3. Child Healthcare and Feeding Practice

Nearly two-thirds (115 (33.3%)) of children were not exclusively breastfed for the first six months of age. About 67 (19.4%) of children have met the minimum acceptable diet (Table 5).

### 3.4. Percentage of Weekly Food Groups' Consumption

**Figure 1:**
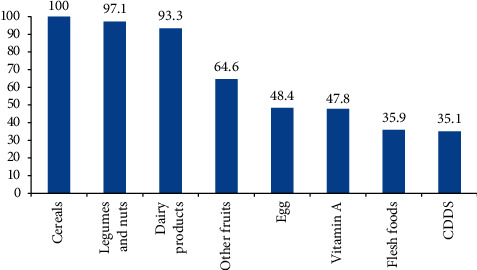
Percentage of children, who consumed the seven food groups one week before the survey, in Golina district, Northeast Ethiopia, 2017.

All of the children consumed cereals for one week before the survey. But only 35.9% of them consumed flesh foods. Children meeting the dietary diversity score was 35.1% (Figure 1).

### 3.5. Infant and Young Child Feeding Practice

**Figure 2:**
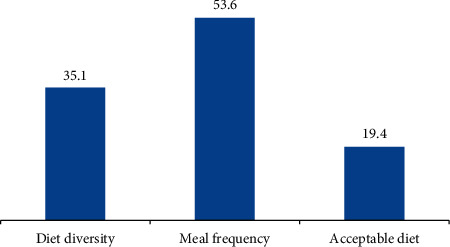
Percentage of infant and young child feeding practice among children aged 6–23 months in Golina district, Northeast Ethiopia (*n* = 345).

Among all children, only 19.4% of them met the minimum acceptable diet (Figure 2).

### 3.6. Factors Associated with Minimum Child Dietary Diversity Score of Children

Child dietary diversity score had no significant association with place of delivery, birth order, maternal residence, child immunization status, and food taboo (Table 6). Children whose mothers attended formal education were 3.042 times (AOR = 3.042 (1.312–7.052)) more likely to meet the minimum optimal dietary diversity score than children whose mothers not attended secondary and above educational status. Children whose mothers were underweight and/or overweight were 51.2% (AOR = 0.488, 95% CI, (0.259–0.918)) and 68.10% (AOR = 0.319, 95% CI, (0.119–0.855)) less likely to meet the optimum minimum dietary diversity score than children whose mothers were with normal BMI, respectively.

## 4. Discussion

In this study, children who met the minimum dietary diversity score were 35.1%, (95% CI: (30%–40%)). This figure is higher than that reported in studies done in Ethiopia such as Arsi Negele district (18.8%) [27], Gorche district (10.6%) [25], and Dangila town (12.6%) [28]. It is also higher than that reported in African countries such as Tanzania (32.58%) and Uganda (29.5%) [30]. But it was lower than those reported in other studies done in Ghana (51.4%) [31], China (63.2%) [32], Indonesia (68.4%) [26], and Kenya (39.06%) [30]. The reason might be due to the difference in the data collection time, awareness creation by health policy systems, media exposure, and community child feeding practice.

This study revealed that maternal nutritional status (BMI) was associated with meeting the minimum dietary diversity score of children. This finding could be explained by the good nutritional status of the mother which might be due to her improved feeding practice, which could be a proxy for her child's good feeding practice. This might be also influenced by decision making and media exposure [28]. This might be also due to wealth index, place of delivery, maternal age, and maternal employment [30].

Maternal education was found to be associated with meeting the child's minimum dietary diversity score. This is in line with studies done in Ethiopia such as Dangilla [28] and African countries (Nigeria [33], Kenya, Uganda, and Tanzania [30]), and Asian country, Nepal [34]. This might be explained by the fact that as the mother's educational level increases, her knowledge, attitude, and practice towards dietary feeding practice might be improved. Also, in addition, as the mother's educational level increases, she might have antenatal care follow-up and media exposure, by which she could get nutritional counseling and improve her child feeding practice [28, 30].

In conclusion, maternal nutrition status and maternal education status were associated with child dietary diversity scores. This study revealed that children who met the minimum dietary diversity score were few. Therefore, increased emphasis on the importance of the education of girls (future mothers) and nutrition counseling for girls/women who received little education on ways to improve the family and child dietary feeding practice is needed.

## Figures and Tables

**Table 1:** Sample size calculation to assess the level of CDDS with the assumption of 95% CI, 5% margin of error, and 5% none response allowance.

Percentage of minimum child dietary diversity score (%)	Reference no.	Minimum final sample size
10.6	[25]	153
68.4	[26]	347
18.8	[27]	247
12.6	[28]	177
30.4	[29]	342

**Table 2:** Sample size calculation to identify factors associated with CDDS using Epi Info (double population proportion formula).

Variables	CI (%)	Power (%)	Ratio (unexposed to exposed)	Outcome in the unexposed group (%)	Outcome in the exposed group (%)	No response obtained (%)	Sample size
Maternal education [28]	95	80	4.56	8.4	25.6	5	268

Index child age [28]	95	80	1.15	6.8	18.3	5	307

**Table 3:** Socioeconomic and demographic characteristics of study participants by the minimum dietary diversity score of children, in Golina district, Northeast Ethiopia, 2017 (*n* = 345).

Variable	Category	Total, *n* (%)	Minimum child dietary diversity score (MCDDS)		*P* value
			Meet, *n* (%)	Not meet, *n* (%)	
Maternal religion	Muslim	315 (91.3)	106 (87.6)	209 (93.3)	0.073
	Others^*∗*^	30 (8.7)	15 (12.4)	15 (6.7)	

Maternal ethnicity	Afar	284 (82.3)	92 (76)	192 (85.7)	0.024
	Others^*∗∗*^	61 (17.7)	26 (24)	32 (14.3)	

Maternal age	15–24	95 (27.5)	39 (32.2)	56 (25.0)	0.338
	25–34	195 (56.5)	65 (53.7)	130 (58.0)	
	≥35	55 (16)	17 (14.1)	38 (17.0)	

Maternal education status	No formal education	254 (73.6)	74 (62.2)	180 (80.4)	<0.0001
	Primary	35 (10.1)	16 (13.2)	19 (8.5)	
	Secondary and above	56 (16.3)	31 (25.6)	25 (11.1)	

Maternal occupation	Government employee	41 (11.9)	24 (19.8)	17 (7.6)	<0.0001
	Pastoralist	216 (62.6)	65 (53.7)	151 (67.4)	
	Housewife	62 (18)	17 (14.0)	45 (20.1)	
	Others^*∗∗∗*^	26 (7.5)	15 (12.3)	11 (4.9)	

Maternal residence	Urban	81 (23.5)	52 (23.2)	29 (24)	0.875
	Rural	264 (76.5)	172 (76.8)	92 (76)	

Index child sex	Male	165 (47.8)	48 (39.7)	117 (52.2)	0.026
	Female	180 (52.2)	73 (60.3)	107 (47.8)	

Index child age	6–8 months	53 (15.4)	26 (21.5)	27 (12.1)	0.025
	9–11 months	77 (22.3)	20 (16.5)	57 (25.4)	
	12–23 months	215 (62.3)	75 (62.0)	140 (62.5)	
Maternal age (in completed years)		Mean ± SD		27.97 ± 5.43	
Family size		Mean ± SD		5.81 ± 2.13	
Index child age (in completed month)		Mean ± SD		13.76 ± 4.57	

^*∗*^Orthodox, Protestant, and Catholic. ^*∗∗*^Amhara and Oromo. ^*∗∗∗*^Student, merchant, and daily laborer.

**Table 4:** Maternal healthcare and nutritional status characteristics by the minimum dietary diversity score of children, in Golina district, Northeast Ethiopia, 2017 (*n* = 345).

Variables	Category	Total, *n* (%)	MCDDS		*P* value
			Meet, *n* (%)	Not meet, *n* (%)	
Maternal ANC follow-up^*∗*^	Yes	143 (41.5)	43 (35.5)	100 (44.6)	0.101
	No	202 (58.5)	78 (64.5)	124 (55.4)	

Birth place	Home	267 (76.8)	93 (76.9)	174 (77.7)	0.862
	Health institution	78 (23.2)	28 (23.1)	50 (22.3)	

Nutrition counseling and health education^*∗∗*^	Yes	82 (23.8)	34 (28.1)	48 (21.4)	0.165
	No	263 (76.2)	87 (71.9)	176 (78.6)	

Maternal BMI (kg/m^2^)	<18.5	73 (21.2)	19 (15.7)	54 (24.1)	0.012
	18.5–24.9	32 (9.3)	6 (5.0)	26 (11.6)	
	≥25	240 (69.5)	96 (79.3)	144 (64.3)	

^*∗*^Mother of the index child making ANC follow-up at least once.^*∗∗*^Getting counseling and education regarding maternal feeding practice (during pregnancy and lactation) and on child feeding practice.

**Table 5:** Child healthcare and feeding-related characteristics by the minimum dietary diversity score of children, in Golina district, Northeast Ethiopia (*n* = 345).

Variables	Category	Total, *n* (%)	MCDDS		*P* value
			Meet, *n* (%)	Not meet, *n* (%)	
Exclusive breastfeeding for the first six months	Yes	115 (33.3)	50 (41.3)	65 (29)	0.021
	No	230 (66.7)	71 (58.7)	159 (71)	

Food taboo for children^*∗*^	Yes	14 (4.1)	2 (1.7)	12 (5.4)	0.096
	No	331 (95.9)	119 (98.3)	212 (94.6)	

Minimum meal frequency (MMF)	Meet	185 (53.6)	67 (55.4)	118 (52.7)	0.632
	Not meet	160 (46.4)	54 (44.6)	106 (47.3)	

Minimum acceptable diet (MAD)	Meet	67 (19.4)	67 (55.4)	0 (0)	<0.0001
	Not meet	278 (80.6)	54 (44.6)	224 (100)	

Immunization status of the child (at least one type)	Yes	170 (49.3)	62 (51.3)	108 (48.2)	0.592
	No	175 (50.7)	59 (47.7)	116 (51.8)	

Birth order of the child^*∗∗*^	≤3	174 (50.4)	71 (58.7)	103 (46)	0.047
	4–5	93 (22.6)	24 (19.8)	69 (30.8)	
	≥6	78 (27)	26 (21.5)	52 (23.2)	

^*∗*^Any type of food which is strictly prohibited for children either culturally or religiously. ^*∗∗*^The order of the index childbirth from all successive births.

**Table 6:** Factors associated with child dietary diversity score in Northeast Ethiopia (*n* = 345).

Variables/Category	Minimum CDDS		COR (95% CI)	AOR (95% CI)
	Meet (≥4)	Not Meet (≤3)		
Maternal education status				
No formal education	74 (61.16)	180 (80.36)	1	1
Attended primary	16 (13.22)	19 (8.48)	2.048 (0.999–4.200)	1.877 (0.807–4.370)
Secondary and above	31 (25.62)	25 (11.16)	3.016 (1.668–5.454)	3.042 (1.312–7.052)

Maternal BMI in (kg/m^2^) status				
Normal	19 (15.7)	54 (24.11)	1	1
Underweight	6 (4.96)	26 (11.61)	0.528 (0.295–0.946)	0.488 (0.259–0.918)
Overweight	96 (79.34)	144 (64.28)	0.346 (0.137–0. 873)	0.319 (0.119–0.855)

The variables entered into the model was maternal educational status, maternal marital status, maternal BMI status, number of under-five children at the household, nutrition counseling during antenatal care follow-up, household decision making power, livestock, index child sex, maternal ethnicity, family size, maternal age, child age, and religion.

## Data Availability

The datasets analyzed during the current study are available from the corresponding author upon reasonable request.

## Abbreviations

AOR: Adjusted odds ratio
BCG: Bacillus Calmette–Guerin
BMI: Body mass index
CI: Confidence interval
CDDS: Child dietary diversity score
EDHS: Ethiopian Demographic and Health Survey
FAO: Food and Agriculture Organization
MDDS: Minimum dietary diversity score
MMF: Minimum meal frequency
MUAC: Mid-upper arm circumference
SPSS: Statistical Package for Social Science
UNICEF: United Nations Children's Fund
WHO: World Health Organization.
